# Small Intestinal and Mesenteric Multiple Gastrointestinal Stromal Tumors Causing Occult Bleeding

**DOI:** 10.1155/2016/5137975

**Published:** 2016-02-16

**Authors:** Tolga Dinc, Selami Ilgaz Kayilioglu, Ahmet Erdogan, Erdinc Cetinkaya, Ozgur Akgul, Faruk Coskun

**Affiliations:** Ankara Numune Training and Research Hospital, Department of General Surgery, 06100 Ankara, Turkey

## Abstract

Gastrointestinal stromal tumors are the meseancymal neoplasms which may involve any part of gastrointestinal tract. C-Kit and platelet derived factor receptor alpha polypeptide are believed to be responsible for the genetic basis. This case presentation aimed to discuss the diagnostic and therapeutic modality of multiple small intestinal, omental, and mesenteric GISTs with different sizes which caused occult bleeding in a 43-year-old male patient.

## 1. Introduction

Gastrointestinal stromal tumor (GIST) was first described by Mazur and Clark in 1983. It is a rare tumor that may involve all parts of gastrointestinal tract [1]. It is believed that mutations of c-Kit protooncogene and platelet derived growth factor receptor alpha polypeptide underlie the genetic basis of the disease. These mutations cause uncontrolled proliferation of Cajal cells by increasing tyrosine kinase activity [2]. GISTs constitute 0.2% of all gastrointestinal tumors and they are mostly located in stomach. Some rare forms may be located in small intestine, omentum, and peritoneum. Only 10–30% of them show malignant transformation [3, 4]. This case presentation aimed to discuss, in the light of the literature, the diagnostic and therapeutic modality of multiple small intestinal, omental, and mesenteric GISTs with different sizes which caused occult bleeding in a young male patient.

## 2. Case Presentation

**Figure 1:**
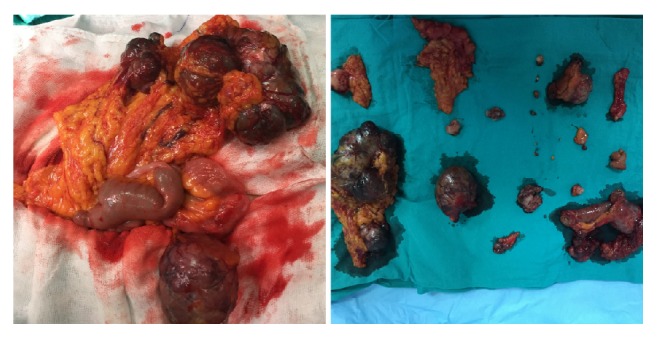
Peroperative and postresection images of the lesions.

A 43-year-old male patient who was admitted to emergency department with abdominal pain, nausea, palpitation, and fatigue was describing a vague pain in the right lower quadrant of abdomen. He had no concomitant disease other than a previous diagnosis of iron deficiency anemia. He had been receiving oral iron preparations. His family history revealed nothing abnormal. On physical examination, conjunctiva was found to be pale. Blood pressure was measured as 100/70 mmHg, heart rate was 110/min., and measured body temperature was within normal limits. On abdominal examination, a 5 cm sized mobile mass was palpated in the right lower quadrant. Complete blood count showed hemoglobin level of 9.2 g/dL and hematocrit of 27.5%, while white blood cell and platelet counts were normal. Since abdominal ultrasonographic (USG) examination demonstrated a hypoechoic right lower quadrant lesion with a size of about 6 cm which had vascularisation on color Doppler ultrasonographic examination, the patient was referred to hematology outpatient clinic for the investigation of the etiology of intraabdominal lymphadenopathy and anemia. The patient in whom performed endoscopic interventions detected nothing pathological underwent computed tomography (CT) which demonstrated semisolid mass lesions with lobulated margins and necrotic centers, which were 3 cm in the middle of the abdomen, 5 cm in the left upper quadrant, 5 cm in mesentery adjacent to superior mesenteric artery, and 6 cm in the right lower quadrant (Figure 1). Tumor markers were found to be normal. The patient was referred to the department of general surgery with the diagnosis of intraabdominal mass.

**Figure 2:**
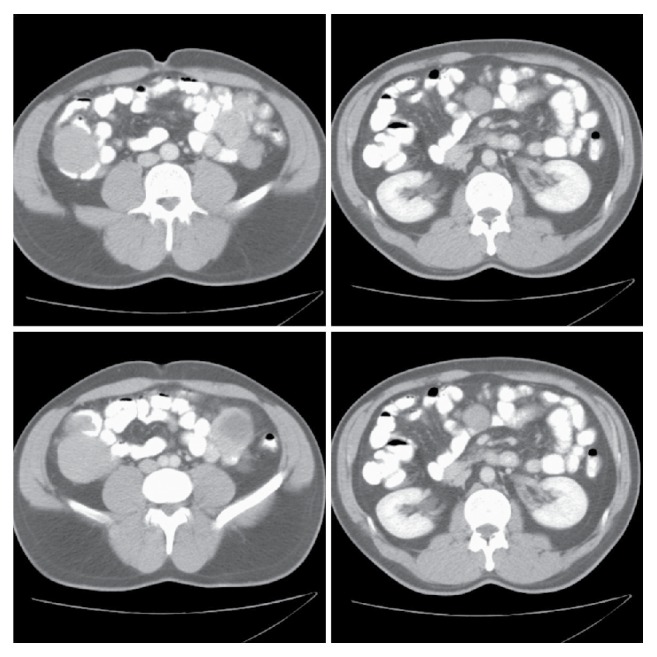
Computed tomography images of the lesions.

The patient was taken to operation after necessary preoperative arrangements were made. Laparotomy with midline incision was performed. Abdominal exploration revealed semisolid mass lesions which were related to intestines, had lobulated margins, and were measured as 4, 5, and 6 cm, being 80, 100, and 180 cm away from treitz ligament, respectively (Figure 2). In addition, there were multiple lesions with different sizes on omentum and mesentery. Peritoneum, liver, and other organs were intact. Three masses adjacent to the intestines were excised. Tumors in the other locations were resected with safe surgical margins. In addition, diverticulectomy was performed in the patient with Meckel diverticulum 4 cm in size; prophylactic appendectomy was also carried out. A drain was placed in the abdomen following bleeding control and anatomic layers were then closed and the operation was terminated.

No problems were confronted during postoperative period. Bowel movements started on the 3rd postoperative day and oral feeding was started and drain was removed. The patient was discharged from the hospital with normal vital signs on the 5th postoperative day. His medical treatment was arranged to include iron preparations, proton pump inhibitor, and oral antibiotic.

Histopathological analysis of the lesions was reported as gastrointestinal stromal tumor. CD117 (c-Kit) was highly positive in more than 75% of the cells. S100 and Vimentin staining were found positive in immunohistochemical analysis, whereas CD34, SMA, Desmin, and Pancreatin were found negative. The patient was referred to relevant clinic for imatinib treatment.

## 3. Discussion

Gastrointestinal stromal tumor is a very rare neoplasm of digestive system with an annual incidence of 1.5/100000 [5]. GIST is a mesenchymal tumor of digestive system and may involve any region from mouth to anus. 90% of the patients are over the age of 40 and the peak incidence occurs between the ages 60 and 65. Its prevalence is slightly higher in males, while it is not affected by geographic region and ethnicity [2]. Common locations of the disease are stomach in 60–70%, small intestine in 20–30%, and esophagus, colon, and rectum in 5–15%; omentum, peritoneum, and mesentery are rarely involved [4]. The most common sites for metastasis are liver and peritoneum, while lymph node involvement is a quite rare condition [4].

It is quite difficult to make preoperative diagnosis of GISTs other than gastric and colorectal forms. Most of the patients are asymptomatic and present with nonspecific symptoms. Failure to visualize small intestine by endoscopic procedures, tendency of the lesion to demonstrate exophytic extension, and limitations in the evaluation of small intestine by CT under emergency conditions have an impact on the difficulty of the diagnosis [6, 7]. GISTs causing bleeding have been described in the literature [8, 9]. Our case also had occult bleeding; he had been referred to hospitals for many times with the symptoms of anemia; he had been repeatedly examined and tested but no diagnosis could be achieved. He had received medical treatment for iron deficiency anemia. Patients with GIST may have bleeding. Bleeding is more common in small intestinal GISTs when compared to gastric, colonic, and rectal GISTs [7]. The origin of bleeding in small intestinal GISTs has been thought to be ulcerated tumoral mucosa, even if it is extraintestinal [7, 10]. Moreover, since stromal collagen is less in amount in the patients with GIST, it is suggested that these patients have thin-walled vessels which are prone to bleeding [11]. Although it is rare, GIST should be considered in the patients presenting with anemia in which the etiology cannot be explained by colonoscopy and endoscopy.

Radiological examination, especially careful evaluation of abdominal CT, is very important in making the diagnosis of the disease. CT gives information on the localization and size of tumor, extension to liver, and peritoneal involvement. It is the most effective imaging modality in the diagnosis of GIST [12]. However, necrotic areas in the center of tumor may not be differentiated from cystic degeneration and abscess, which may lead to misdiagnosis [13]. CT appearance of most GISTs is an exophytic lesion >5 cm in size which is well demarcated and has lobulated margins, necrosis, or hemorrhage at the center but no calcification [10, 14, 15]. CT examination of our case revealed 3 separate mass lesions which were 3–6 cm in size and originating from small intestine and a mesenteric lesion 5 cm in size. All lesions had necrotic areas in the center and lobulated margins. CT images of our patient were consistent with CT images of other patients with GIST reported in the literature.

The main point of the treatment is surgical removal of the tumor, which is best to achieve cure. It is important to preserve pseudocapsule of tumor, while extensive intestinal resection is not necessary [16]. Preservation of capsule is important in that it prevents intraabdominal seeding during surgery [17]. Rupture of tumor negatively affects the prognosis [15, 18]. In our patient, tumor was removed leaving negative surgical margins and the integrity of pseudocapsule was not disrupted.

Mesenteric GISTs are quite rarely seen. c-Kit and platelet derived growth factor receptor alpha mutations can be detected in most of the cases. Presence of any of these mutations is important for diagnosis [19].

Since GIST generally does not spread to lymph nodes, lymphadenectomy is usually not required in surgical treatment of GIST [16, 17]. In our case, no lymphadenopathy was detected peroperatively and lymph node dissection was not performed.

Literature data suggest that tumors smaller than 10 cm are less likely to metastasize, have lower mitotic index and no intraperitoneal spread, and may be treated by R0 surgery [20, 21]. Our case also had tumors less than 10 cm in size, which were excised according to the above criteria.

## 4. Conclusion

Small intestinal GIST should be considered in the patients presenting with nonspecific abdominal pain and the symptoms of anemia. This disease should be remembered in the patients with normal endoscopic examinations, in whom large mesenteric lymph nodes which are conglomerated, have lobulated margins and necrotic centers, and are located adjacent to intestine are present. In addition, it should be kept in mind that it may have multiple origins and various sizes and may involve multiple organs.
